# The role of small RNAs in wide hybridisation and allopolyploidisation between *Brassica rapa* and *Brassica nigra*

**DOI:** 10.1186/s12870-014-0272-9

**Published:** 2014-10-19

**Authors:** Muhammad Awais Ghani, Junxing Li, Linli Rao, Muhammad Ammar Raza, Liwen Cao, Ningning Yu, Xiaoxia Zou, Liping Chen

**Affiliations:** Department of Horticulture, College of Agriculture and Biotechnology, Zhejiang University, Yuhangtang Road No.866, Hangzhou, 310058 Zhejiang Province P. R. China; Key Laboratory of Horticultural Plant Growth, Development, and Biotechnology, Agricultural Ministry of China, Hangzhou, 310058 P. R. China

**Keywords:** Wide hybridisation, Allopolyploidisation, DNA methylation, Small RNAs

## Abstract

**Background:**

An allopolyploid formation consists of the two processes of hybridisation and chromosome doubling. Hybridisation makes a different genome combined in the same cell, and genome “shock” and instability occur during this process, whereas chromosome doubling results in doubling and reconstructing the genome dosage. Recent studies have demonstrated that small RNAs, play an important role in maintaining the genome reconstruction and stability. However, to date, little is known regarding the role of small RNAs during the process of wide hybridisation and chromosome doubling, which is essential to elucidate the mechanism of polyploidisation. Therefore, the genetic and DNA methylation alterations and changes in the siRNA and miRNA were assessed during the formation of an allodiploid and its allotetraploid between *Brassica rapa* and *Brassica nigra* in the present study.

**Results:**

The phenotypic analysis exhibited that the allotetraploid had high heterosis compared with their parents and the allodiploid. The methylation-sensitive amplification polymorphism (MSAP) analysis indicated that the proportion of changes in the methylation pattern of the allodiploid was significantly higher than that found in the allotetraploid, while the DNA methylation ratio was higher in the parents than the allodiploid and allotetraploid. The small RNAs results showed that the expression levels of miRNAs increased in the allodiploid and allotetraploid compared with the parents, and the expression levels of siRNAs increased and decreased compared with the parents *B. rapa* and *B. nigra*, respectively. Moreover, the percentages of miRNAs increased with an increase in the polyploidy levels, but the percentages of siRNAs and DNA methylation alterations decreased with an increase in the polyploidy levels. Furthermore, qRT-PCR analysis showed that the expression levels of the target genes were negatively corrected with the expressed miRNAs.

**Conclusions:**

The study showed that siRNAs and DNA methylation play an important role in maintaining the genome stability in the formation of an allotetraploid. The miRNAs regulate gene expression and induce the phenotype variation, which may play an important role in the occurrence of heterosis in the allotetraploid. The findings of this study may provide new information for elucidating that the allotetraploids have a growth advantage over the parents and the allodiploids.

**Electronic supplementary material:**

The online version of this article (doi:10.1186/s12870-014-0272-9) contains supplementary material, which is available to authorized users.

## Background

Wide hybridisation and polyploidisation is a common phenomenon in plant evolution that results in the formation of new species [1-4]. Wide hybrids often exhibit more vigorous growth than their parents, and this effect is mainly demonstrated by increases in drought tolerance, pest resistance, flowering time, organ size and biomass, among other factors [5-7]. Furthermore, polyploids show novel traits that are not present in their diploid progenitors [8]. For example, allotetraploid cotton (genomes: AADD) produces more abundant and higher-quality fibres, and this effect is derived from their AA and DD extant diploid species [9]. However, wide hybrids also exhibit the disadvantage of infertility, as was observed with the wide hybridisation combination of *B. rapa* and *B. oleracea* [10]. However, despite this finding, the growth and adaptability advantages of polyploidy have always been a puzzling phenomenon, and the underlying molecular mechanisms are among the most interesting subjects in plant breeding.

Allopolyploid formation consists of two processes, namely, hybridisation and chromosome doubling. Hybridisation involves a different genomic combination in the same cells and genomes experience “genomic shock”, whereas chromosome doubling doubles and restructures the genome dosage [11,12]. Recent studies have shown that small RNAs, particularly the 24-nt siRNAs, play an important role in genome reconstruction and stabilisation [13]. The role of 24-nt siRNAs is primarily reflected in two aspects. The first aspect is the modification of transposons and repetitive sequences for the maintenance of genome stability, which is mediated by RNA-dependent DNA methylation, and the other aspect is the *cis*-regulation of gene expression via the transposon gene fragment in the gene near the region (such as the promoter expression area) [14-18]. The reduction of siRNAs in F_1_ may induce phenotypic and genomic instability in *Arabidopsis* allotetraploids [13,19]. In interspecific hybrids of *Arabidopsis*, the siRNA populations underwent rapid changes in the F_1_ allotetraploid, but stability was maintained in the F_7_ allotetraploid, in which the DNA and chromatin were significantly modified [13]. However, in wheat, the number of 24-nt siRNA transcripts significantly decreased in the hexaploid compared with that obtained in the parents, and this decrease was accompanied by a decrease in the DNA chain CpG island methylation levels [20]. Thus, these studies concluded that a decrease in the DNA methylation levels may be one of the causes of genomic instability in allopolyploids during the early stage [21,13]. This phenomenon of changes to the siRNAs with an increase in the polyploidy level suggests that siRNAs may play a key role in genomic reconstruction and stability after chromosome doubling. However, little is known regarding how siRNAs play this role during wide hybridisation (with different genome combined) and chromosome doubling (the doubling of the genome dosage).

miRNAs are the primary mediators of the trans-regulation of gene expression [22]. Gene silencing mediated by miRNAs is an important strategy used at the post-transcriptional level of gene regulation [23,24]. Furthermore, miRNAs are conserved in evolution but become active in polyploidisation [25,13]. Changes in the miRNA expression levels can affect the expression of the target genes, and this effect is considered to be one of the main causes that results in the phenotypic variation of the polyploidy [21,26,27,13]. In *Arabidopsis*, the number of miRNAs in allotetraploids is higher than those observed in their autotetraploid parents [13]. A similar phenomenon was also found in the synthesis of hexaploid wheat [20]: the number of miRNAs increased with an increase in the ploidy level, and this effect was not associated with the gene dosage balance hypothesis. This phenomenon showed that other mechanisms, such as *cis*-epigenetic regulation, may exist. Thus, small RNAs, which are a product of non-coding RNAs, are involved in regulating gene expression and have become an important factor of gene expression during allopolyploidisation. However, little is known regarding the changes in miRNAs and their regulation of gene expression and phenotypic variation during wide hybridisation and chromosome doubling, and these data are essential for elucidating the mechanism of heterosis.

In a previous study, we performed wide hybridisation between *B. rapa* (genome: AA, 2n = 20) and *B. nigra* (genome: BB, 2n = 16) and obtained an allodiploid (genome: AB) and allotetraploid (genome: AABB). In addition, we showed that chromosome doubling resulted in higher levels of genetic and phenotypic variation compared with wide hybridisation [28]. In this study, we first analysed the allodiploids and allotetraploids using sequence-related amplified polymorphism (SRAP) and methylation-sensitive amplification polymorphism (MSAP) to determine the differences in the genetic changes and epigenetic alterations between wide hybridisation and chromosome doubling. Second, the allodiploids and allotetraploids were analysed through the high-throughput sequencing of small RNAs to determine how the changes in small RNAs occur during these two processes. Different genomes were combined, the genome dosage was doubled, and the correlation between the siRNA and DNA methylation at different polyploid levels was assessed. Third, the different expression levels of known miRNAs and their target genes were analysed to explore how miRNAs and their target genes affect the different phenotypes of the allodiploids and allotetraploids.

## Results

### Phenotypic analysis of the parents and their wide hybrids

**Figure 1:**
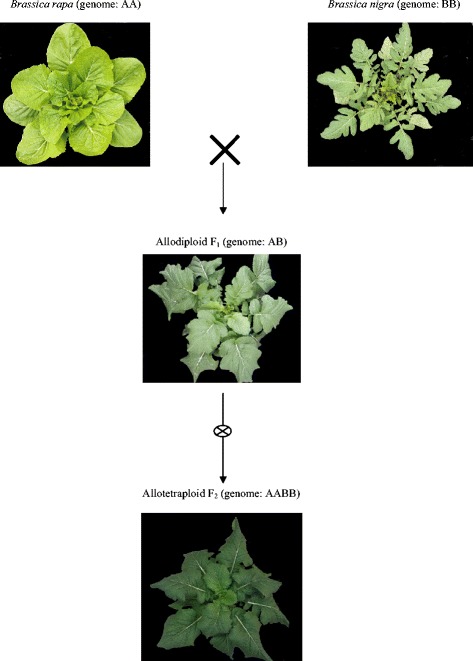
**The layout of the experiment plants; the parents and their allodiploid and allotetraploid.**

**Figure 2:**
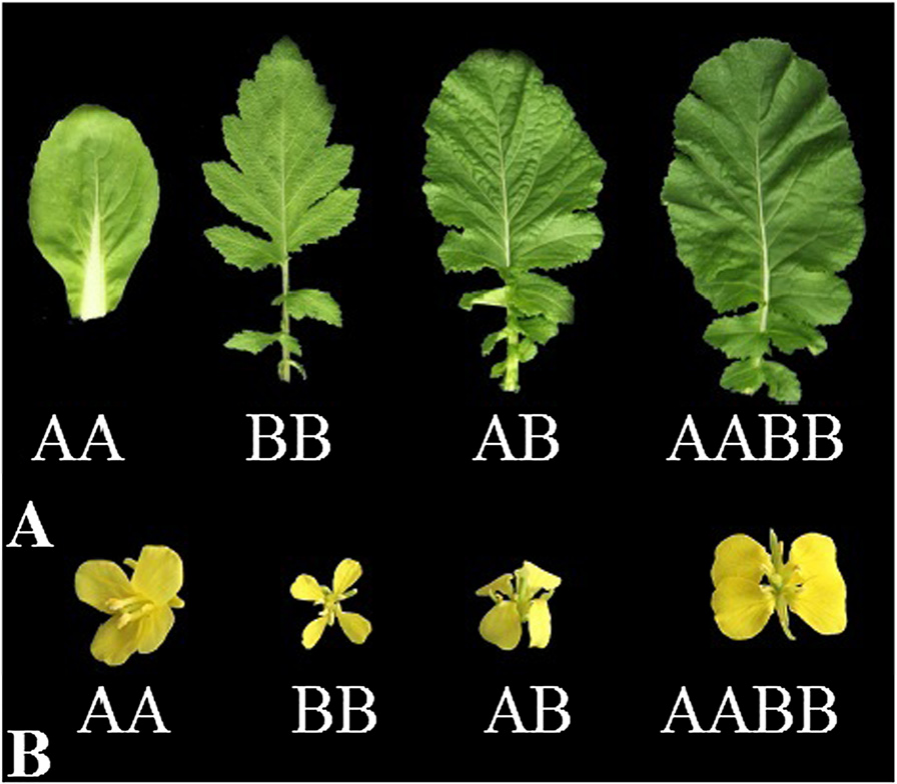
**A. Leaves and B. flower of the parents and their allodiploid and allotetraploid.** Allotetraploids had a high leaf length and flower size compared with their parents and the allodiploids.

In our previous study, the wide hybridisation of *B. rapa* (genome AB) and *B. nigra* (genome AA) was performed, and the allodiploid (genome AB) and allotetraploid (genome AABB) were obtained. We aimed to determine the phenotypic differences between the wide hybrid and their parents. The characteristics of the allodiploid and allotetraploid and their parents were compared (Figures 1 and 2). The results showed that allotetraploids had a high leaf length and flower size compared with their parents and the allodiploids (Figures 1 and 2). In our previous study, we found that allotetraploids had greater vigour than their parents and allodiploids [28]. Thus, allotetraploids had high heterosis compared with their parents and the allodiploids.

### DNA methylation patterns of the parents and their wide hybrids

To elucidate the epigenetic mechanisms related to the processes of hybridisation and polyploidisation, methylation-sensitive amplification polymorphism (MSAP) analysis was used to analyse the parents and their allodiploid (AB) and allotetraploid (AABB). After treatment with double-restriction *EcoR*I/*Msp*I or *EcoR*I /*Hpa*II, the amplified fragments were classified as one of four types: (a) non-methylated in all of the samples, (b) methylated in all of the samples, (c) demethylated in the hybrids compared with the parents, and (d) hyper-methylated in the hybrids compared with the parents (Additional file 1).

**Figure 3:**
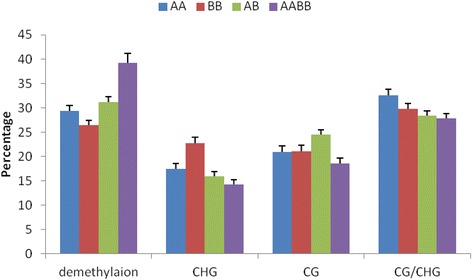
**DNA methylation in the parents and their allodiploid and allotetraploid.**

**Figure 4:**
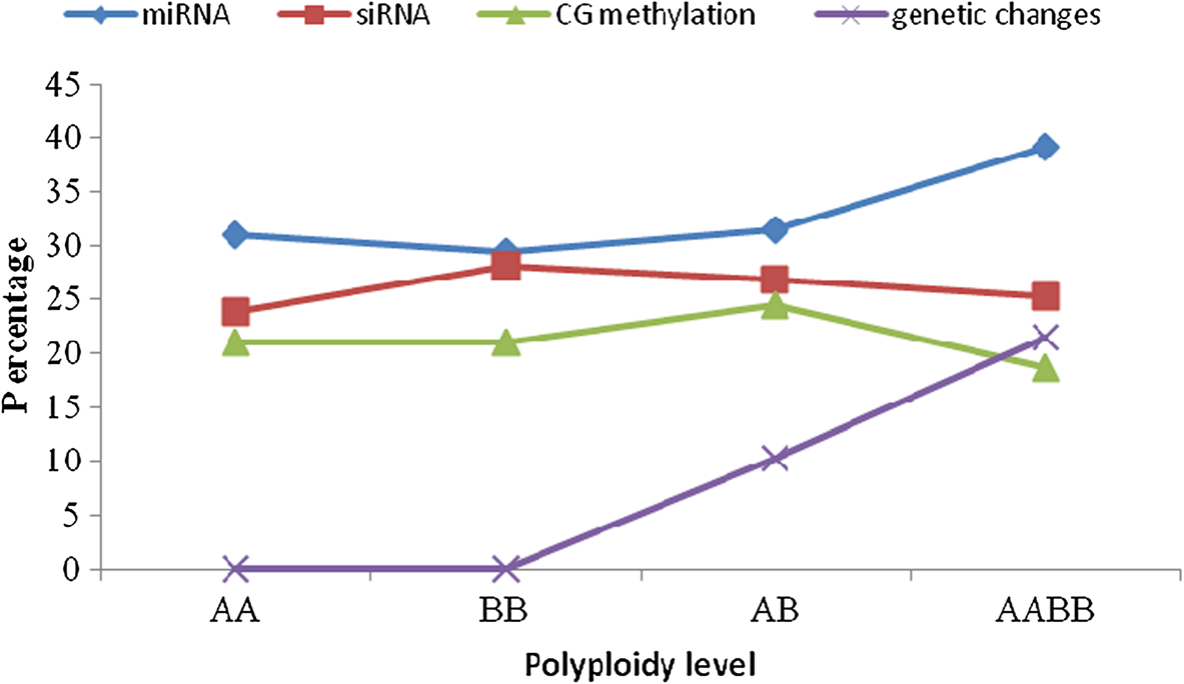
**The percentage of miRNAs, siRNAs, genetic changes, and CG methylation in the parents and their allodiploid and allotetraploid.**

In this study, 1449 reproducible and clear loci were obtained using 36 primer pairs (Additional file 2). These 1449 loci were classified into four major groups (a-d), including 60 categories according to the variation model between the parents and their allodiploids and allotetraploids (Additional file 1). Group A consisted of 12.22% and 12.08% of the monomorphic loci in AB and AABB, respectively. In Group B, 11.94% of the polymorphic loci were specifically found in AB and AABB. Of the loci in Group C, 18.91% and 28.64% of the polymorphic loci were specifically found in AB and AABB, respectively. Compared with the parents, the Group D loci displayed alterations in DNA methylation only in 56.94% and 48.10% of those found in AB and AABB, respectively. There was a significant difference in the methylation patterns between AB and AABB. With respect to the DNA methylation status, the ratios between the allodiploids, the allotetraploids, and their parents demonstrated significant differences (Additional file 1). In addition, the CG methylation was high (24.50%) in AB compared with AABB (Figure 3 and Additional file 1). Thus, the DNA methylation alteration in AB was significantly higher compared with that in AABB. Moreover, the genetic study revealed that two types of the fragments can be used to estimate the genomic changes in the allodiploids and allotetraploids, which have a loss of fragments compared with the parents in addition to novel fragments. The percentages of genetic changes were 10.32% in AB and 21.41% in AABB (Figure 4 and Additional file 3). Thus, the percentage of genetic changes was significantly higher in AABB compared with AB.

### High-throughput sequencing of small RNAs

**Figure 5:**
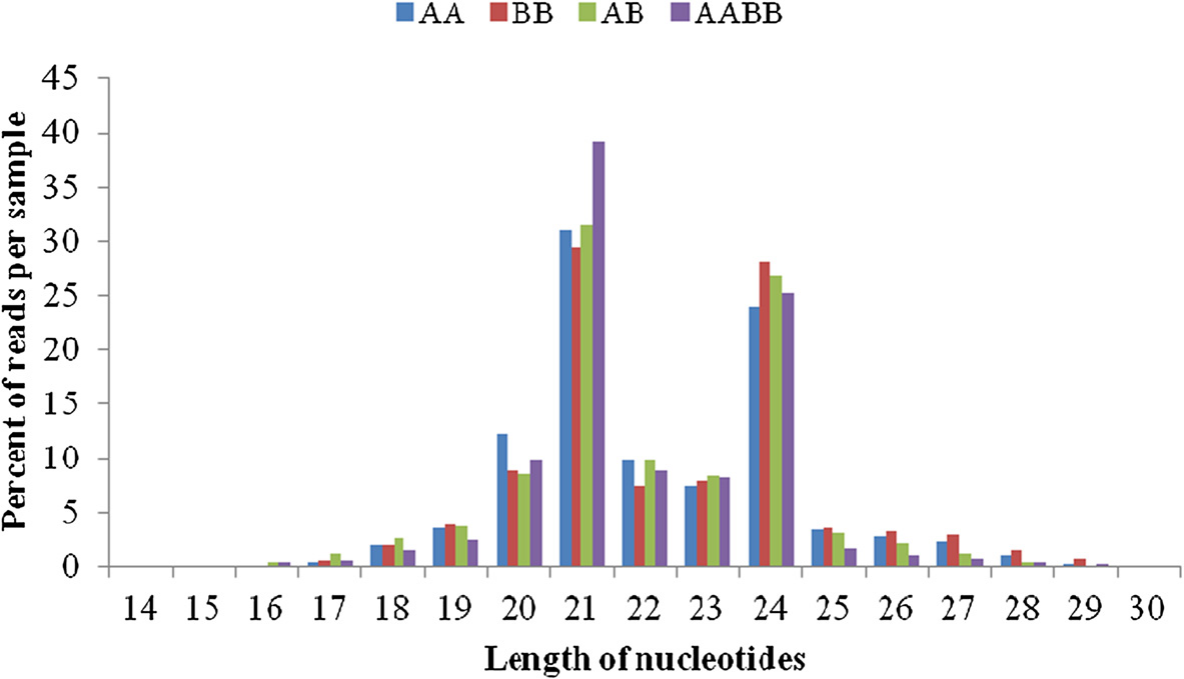
**The length of the small RNAs in the parents and their allodiploid and allotetraploid.**

A small RNA library was prepared for the analysis of the parents and their allodiploids and allotetraploids. Sixty million reads were obtained from the small RNA sequencing of the four libraries (Additional file 4). A total of 41,810,504 reads were obtained, and 41,664,822 of these reads were of high quality and corresponded to 28,426,693 unique sequence tags. The small RNA sizes ranged from 18 to 30 nt, which included the two prominent classes of 21-nt and 24-nt long small RNAs (Figure 5). The 21-nt class corresponded predominantly to miRNAs, whereas the 24-nt class corresponded to siRNAs [29]. The 24-nt long small RNAs were most abundant within the two parental and AB libraries, whereas the 21-nt long small RNAs were most prevalent in the AABB library. Interestingly, the amount of miRNAs relative to the total small RNAs increased with increasing levels of polyploidy: the lowest percentages were 30.9% in AA, 29.34% in BB, 31.44% in AB, and 39.22% in AABB. However, the amount of siRNAs relative to the total small RNA decreased with the increasing levels of polyploidy: the highest percentages were 23.93% in AA, 28.14% in BB, 26.90% in AB, and 25.23% in AABB (Figure 4). Moreover, the total small RNAs demonstrated a high interaction of 81.31% in AA/AB, and the unique small RNAs showed a high level of interaction of 15.34% in AA/AB (Additional file 4). The genome-matched small RNA tags were then clustered into several RNA categories (such as known mRNAs, repeat-associated RNAs, rRNAs, tRNAs, snRNAs, and snoRNAs) in the four libraries (Additional file 4). In addition, a high percentage of small RNAs were sorted as unann RNAs (44.81% in AA, 44.23% in BB, 48.86% in AB, and 53.55% in AABB). The repeat-associated sRNAs were matched based on LTR/Copia: 0, LTR/Copia: 1, LTR/Gypsy: 0, and LTR/Gypsy: 1 in both the unique tags and total tags. Unexpectedly, all four types of repeat-associated sRNAs accumulated in lower levels in AABB compared with AB (Additional file 5). Among the four types, the accumulation of 21-nt sRNAs was higher compared with that of 24-nt sRNAs.

### Known miRNAs

**Figure 6:**
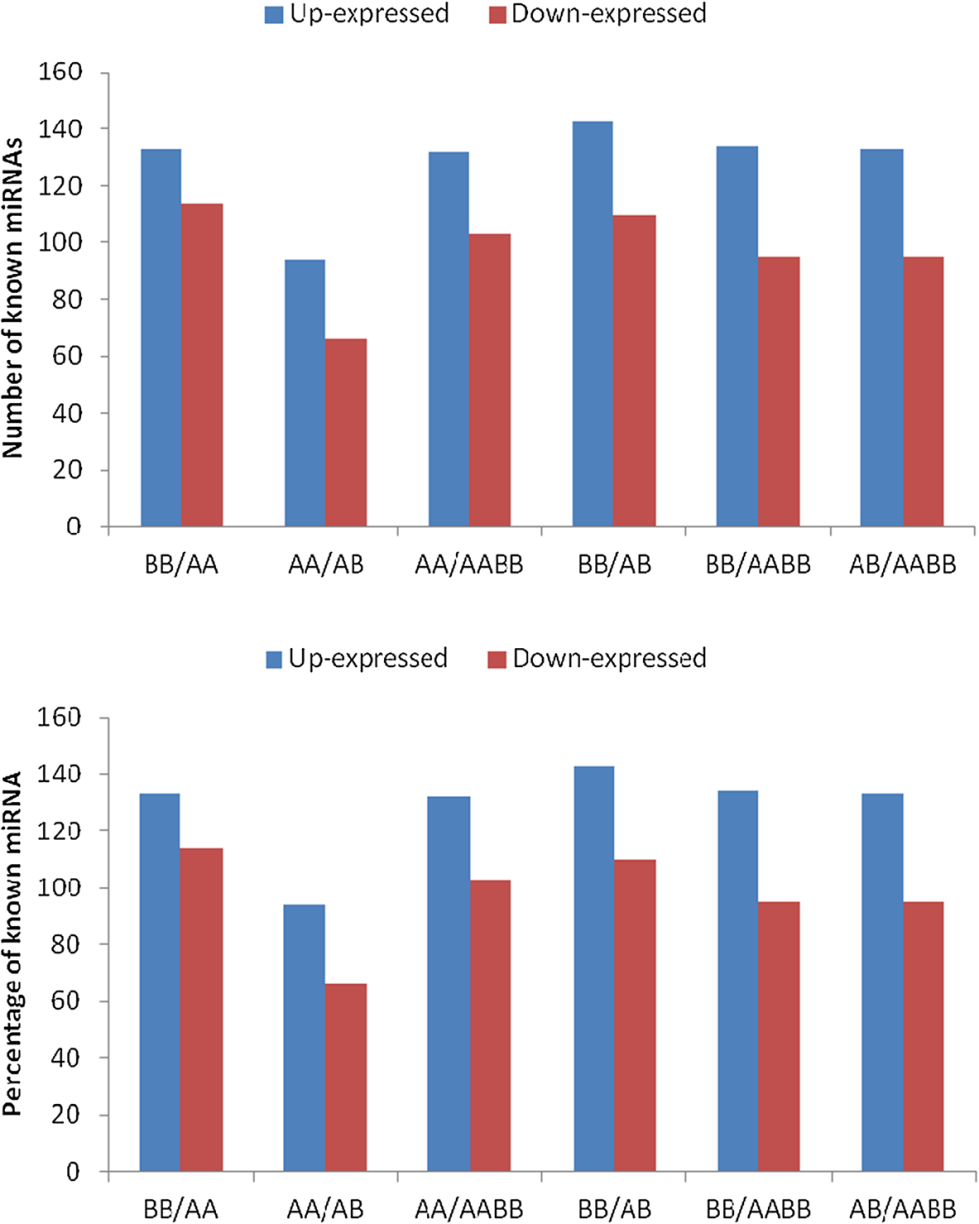
**The expression levels of the known miRNAs in the parents and their allodiploid and allotetraploid.**

To identify conserved miRNAs in the parents and their allodiploids and allotetraploids, small RNAs that were 18–23 nucleotides in length were searched using Blastn against miRBase version V17.0. The 22,954 and 22,716 unique sequences (2,526,482 and 2,176,470 reads) that were found in the AA and BB libraries, respectively, were annotated as miRNA candidates. Totals of 24,143 and 23,061 unique sequences (2,183,129 and 2,524,964 reads) were found in the AB and AABB libraries, respectively (Additional file 4). The expression of known miRNAs in the four samples was demonstrated by plotting the log_2_-ratio (Additional file 6). These results showed 320 differentially expressed miRNAs: 133, 94, 132, 143, 134, and 133 up-regulated miRNAs and 114, 66, 103, 110, 95, and 95 down-regulated miRNAs in BB/AA, AA/AB, AA/AABB, BB/AB, BB/AABB, and AB/AABB, respectively (Figure 6). These findings were significantly different between the four libraries. In our study, further analysis identified totals of 1235, 2967, 1189, and 2651 conserved miRNAs, which belong to 68 plants families, in the AA, BB, AB, and AABB libraries, respectively (Additional file 7).

### Novel miRNAs

**Figure 7:**
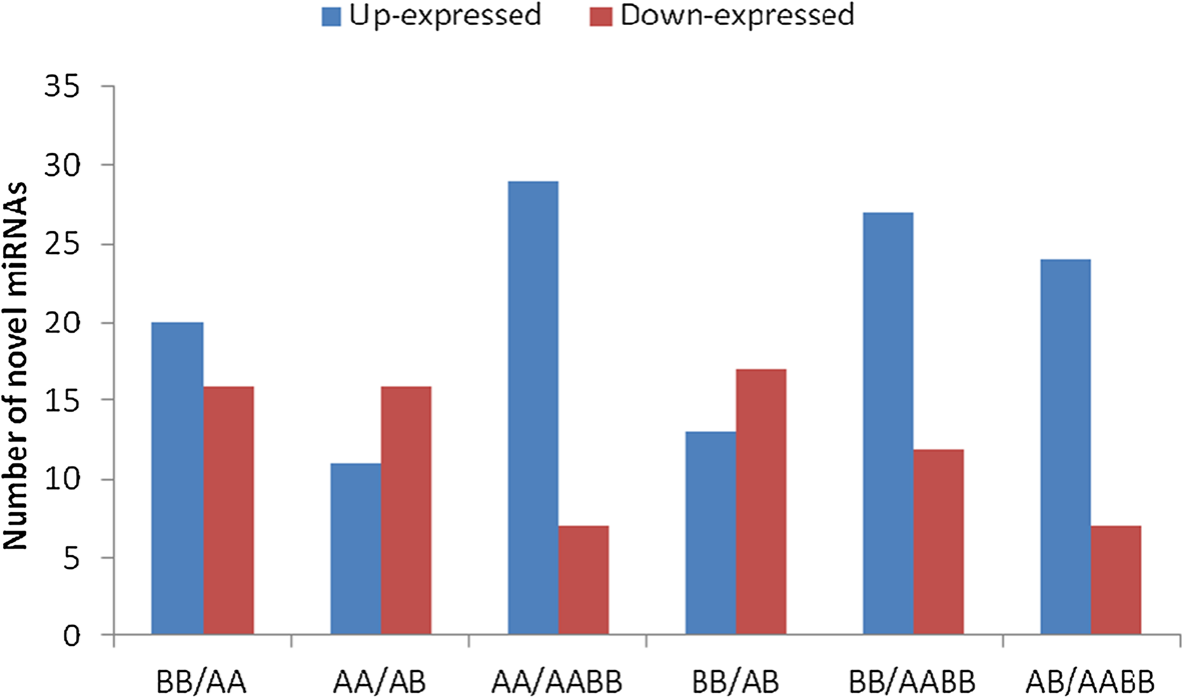
**The expression levels of the novel miRNAs in the parents and their allodiploid and allotetraploid.**

The expression of novel miRNAs in the four samples was demonstrated by plotting the log_2_-ratio (Additional file 6). These results showed 52 novel miRNAs: 20, 11, 29, 13, 27, and 24 up-regulated miRNAs and 16, 16, 7, 17, 12, and 7 down-regulated miRNAs in BB/AA, AA/AB, AA/AABB, BB/AB, BB/AABB, and AB/AABB, respectively (Figure 7). These findings were significantly different between the four libraries.

### qRT-PCR analysis of miRNAs and their target genes

**Figure 8:**
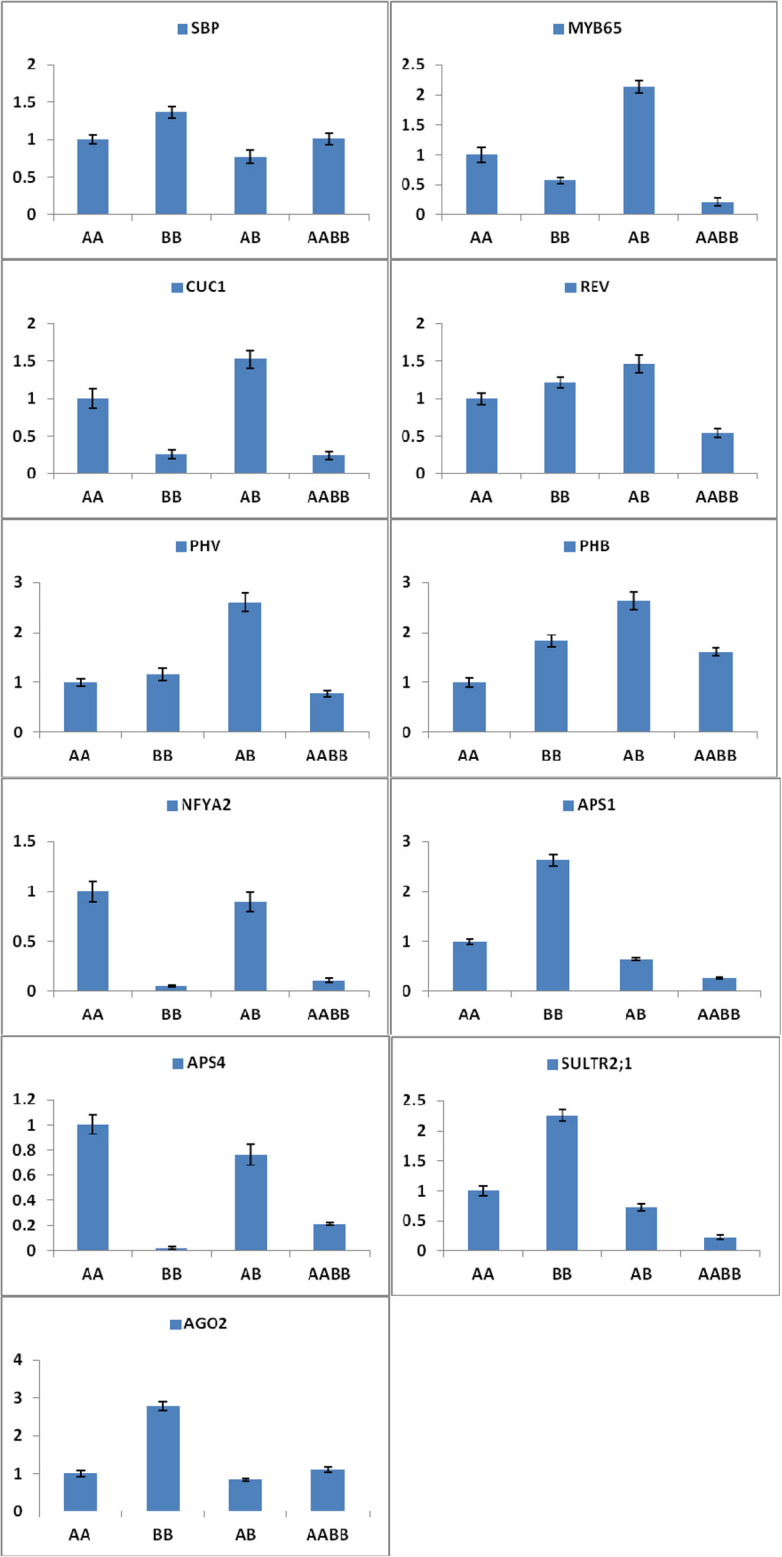
**The expression of miRNA targets in the parents and their allodiploid and allotetraploid.**

**Figure 9:**
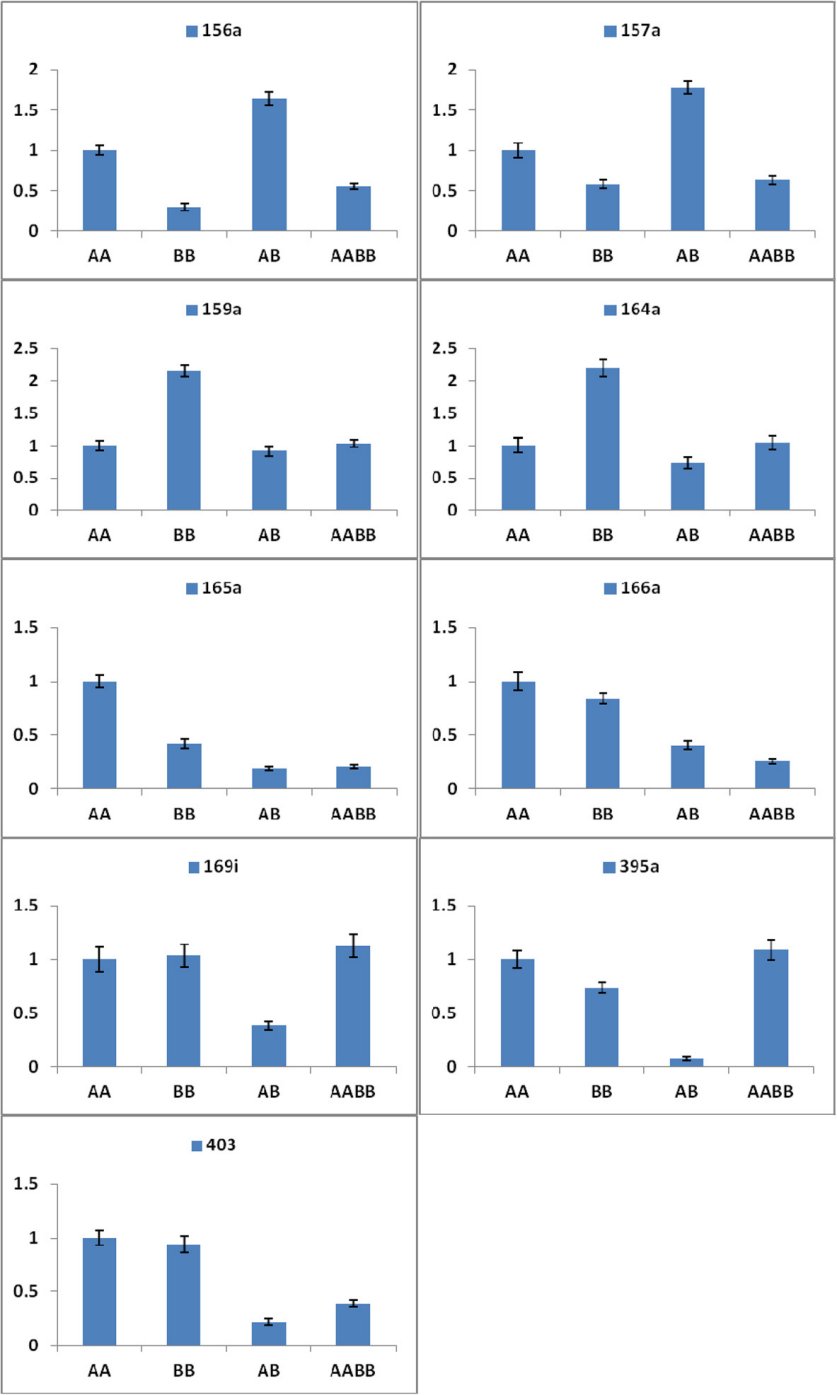
**The expression levels of the miRNAs in the parents and their allodiploid and allotetraploid.**

Many miRNA targets play important roles in the regulation of their expression. The targets of the differentially expressed miRNAs were predicted to elucidate the relationship between functions and phenotypes. The prediction revealed a total of 641 known miRNAs and 3734 target genes: 158, 160, 159, and 164 known miRNAs and 895, 972, 895, and 972 target genes in AA, BB, AB, and AABB, respectively (Additional file 6). Moreover, a total of 68 novel miRNAs and 225 target genes were detected: 11, 17, 14, and 56 novel miRNAs and 49, 53, 20, and 103 target genes in AA, BB, AB, and AABB, respectively (Additional file 6). Furthermore, we performed a quantitative analysis of nine miRNAs and 11 targets genes involved in different vegetative and reproductive functions. Moreover, the expression levels of the target genes (MYB65, CUC1, PHV, PHB, REV, NFYA2, APS1, APS4, and SULTR2:1) were higher in AB compared with AABB. However, the expression levels of SBP and AGO2 were higher in AABB compared with AB (Figure 8). Although the corresponding accumulated abundances of various miRNAs (miR159a, miR164a, miR165a, miR169i, miR395a, and miR403) were lower in AB compared with AABB, the levels of miR156a and miR157a were higher in AB compared with AABB (Figure 9). Moreover, only miR165a, miR166a, and miR395a were not inversely correlated with the targeted genes (REV, PHV, and APS1, SULTR2;1) in AABB and AB, respectively. Thus, the expression levels of the targets were negatively corrected with the abundance of significantly expressed miRNAs in this study.

## Discussion

### Wide hybridisation and polyploidisation showed different patterns of DNA methylation

In this study, the siRNAs and DNA methylation patterns were significantly different between the allodiploid (AB) and the allotetraploid (AABB). DNA methylation was mediated by 24-nt siRNAs, which are derived from repetitive DNA, transposons, and intergenic and genic regions [24,30]. Based on the length distribution of small RNAs, 24-nt siRNAs ranged from 26.90% (AB) to 25.23% (AABB) (Figure 5). The repeat-associated siRNAs were matched on the LTR (retrotransposons) and showed higher levels in AB compared with AABB (Additional file 5). The LTR can be reactivated by hybridisation, as has been demonstrated by several previous studies [31,32]. Thus, a hypothesis may be reached that the extent of retrotransponson activation varies depending on the wide hybridisation. Thus, the regulation of genomic dosage may display different patterns in AB and AABB.

DNA methylation combined with the activation of a transposable element has been proposed as the stabilising mechanism underlying the epigenetic changes mediated by siRNAs during hybridisation and polyploidisation [13,33-35]. In response to genomic shock, the siRNAs maintain genomic stability in allopolyploids [13]. Thus, it can be speculated that siRNAs and DNA methylation are conducive to the maintenance of genome stability in AABB when faced with genomic shock in AB. Moreover, reduced siRNAs levels are largely associated with genes, and genes are associated with altered siRNAs levels and have correlations with changes in the DNA methylation and expression levels [36]. In the present study, the detected siRNAs and DNA methylation were low in the allotetraploids compared with the allodiploids. Thus, it has been proposed that siRNAs play a key role in maintaining the genomic stability of allotetraploids.

### Changes in siRNA and miRNA during wide hybridisation and polyploidisation

siRNAs and miRNAs induce rapid and dynamic changes during the early stage of allopolyploid formation [13]. The most abundant small RNAs found in this study through high-throughput sequencing were identified as miRNAs and siRNAs, as assessed by the composition of miRNAs and siRNAs, which were often 21-nt and 24-nt in length, respectively [23]. The relative amount of small RNAs corresponding to miRNAs increased with an increase in the polyploidy level (Figure 4). Conversely, the relative amount of siRNAs corresponding to transposons decreased with an increase in the polyploidy level (Figure 4). Similarly, Cantu et al. [37] demonstrated that siRNAs corresponding to transposons are observed at lower levels in hexaploids compared with tetraploids. Moreover, the relative amount of epigenetic alterations decreased with an increase in the polyploidy level (Figure 4). Kenan-Eichler et al. [20] also reported that the percentage of siRNAs and epigenetic changes decreased and the miRNA levels increased in hexaploids. Thus, the miRNA expression increased with an increase in the polyploidy level, whereas the siRNA and epigenetic alteration levels decreased with an increase in the polyploidy level. Furthermore, this ploidy dependence was insensitive to genomic composition but was sensitive to dosage, such as that of AB and AABB, which featured the same two genomes (A and B) at varying doses (2× vs. 4×), resulting in their expression of divergent small RNAs profiles.

### The expression of miRNAs and their target genes in the allodiploids and allotetraploids

miRNAs function as negative regulators of gene expression and are known to play markedly expanded roles in a variety of developmental processes affecting meristems, leaves, roots, and inflorescences [38]. The evolutionary conserved miR164, miR165, and miR166 regulate the development of leaves and contribute to the construction of leaf morphology. The overexpression of miR164, miR165 and miR166 reduce the levels of all CUC1, PHB, PHV, and REV genes and increase the development of SAM, which has effects on leaf development [39-42]. Similarly, the miR164a, miR165a, and miR166a levels were high and the CUC1, PHB, PHV, and REV levels were low in AABB compared with AB (Figures 9 and 8). In our previous study, AB and AABB presented significantly different phenotypes with respect to leaf length and width (Figure 2) [28].

In the vegetative to the reproductive phase, the targets of miR156, miR157, miR159 and miR169 participate in the activation of floral meristem identity genes. In *Arabidopsis*, as development proceeds, the decrease in the miR156/miR157 levels and the increase in SPLs in the SAM result in the activation of floral meristem identity genes [43,44]. A high expression level of miR169 is known to have a positive effect on the timing and development of flowers downstream of NFYA2 [42,43,45,46]. Similarly, in our phenotypic study, flower development was high in AABB compared with AB (Figures 2, 9, and 8). Moreover, the overexpression of miR159 results in a delay in flowering, which is associated with a reduction in the levels of MYB [47-49]. In our findings, flower timing was delayed in AABB compared with AB (Figures 9 and 8 and Additional file 8).

A large number of miRNAs from diverse plants have been identified in the response to metal stress [50-52]. Recent studies have shown that miR395 is induced by sulphur starvation and regulates a low-affinity sulphate transporter (SULTR2;1) and three ATP sulphurylases (including APS1 and APS4) [50,53-55]. Furthermore, transgenic plants over-expressing miR395 accumulate more sulphate in the plant shoots, which suggests that miR395 is involved in the regulation of sulphate allocation by targeting APS genes and SULTR2;1 [55]. In our study, AABB plants were found to overexpress miR395 and presented low expression of its target genes (APS1, APS4, and SULTR2;1) compared with AB plants (Figures 9 and 8). These results showed that AABB plants exhibit tolerance to sulphate deficiency and heavy metal stress. Previous studies have shown that AGO2 mRNA is targeted by miR403 [54,56]. Moreover, the regulation of miRNAs and their targets may result in novel phenotypes in allopolyploids [13]. In addition, AABB plants presented a higher level of AGO2 compared with the AB plants (Figure 8). Taken together, the data suggest that miRNAs regulate gene expression and induce phenotype variation, such as heterosis, in allotetraploids.

This study explored the role of small RNAs in wide hybridisation and polyploidisation between *B. rapa* and *B nigra*. The different miRNAs showed different expression levels in allodiploids and allotetraploids, which performed the phenotypic variation. However, some questions remain elusive, including how nonadditively expressed miRNAs and siRNAs affect growth and developmental traits, such as leaf shape, plant stature, biomass, flowering time, and fitness in allodiploids and allotetraploids, and whether and how siRNAs and DNA methylation play roles in this process. Therefore, further studies of the allodiploids and allotetraploids will be necessary.

## Conclusions

This study explored the role of small RNAs in wide hybridisation and allopolyploidisation between *Brassica rapa* (genome: AA) and *Brassica nigra* (genome: BB). When the A genome was crossed with the B genome, siRNA levels increased and decreased relative to their parents; *B. rapa* and *B. nigra*, respectively, while the DNA methylation levels increased relative to their parents. When the genome AB was doubled, the siRNA and DNA methylation levels of the allotetraploid decreased compared with its allodiploid. When the A genome was crossed with the B genome, the miRNA levels increased relative to their parents. When the genome AB was doubled, the miRNA levels of the allotetraploid increased compared with its allodiploid. This result showed that siRNAs, DNA methylation and miRNA play key roles in maintaining the genomic stability through the regulation of small RNA levels. Moreover, most miRNAs were highly overexpressed in the allotetraploid, which might be induced by the heterosis, such as miR159, miR169, and miR164, miR165, and miR166, which have a major role in flower and leaf development in the allotetraploid. Taken together, the findings of this study demonstrated that siRNAs and miRNAs maintain the genomic and phenotypic stability in the allotetraploid. Therefore, the present findings may provide new information for elucidating the effects of small RNAs on the formation of allopolyploidisation.

## Methods

### Plant materials

Wide hybridisation between *B. rapa* (♀, genome: AA) and *B. nigra* (♂, genome: BB) was performed to produce allodiploids (F_1_, genome: AB), and subsequently allotetraploids (F_1_, genome: AABB) were obtained by treating the allodiploids with 0.2% colchicine for 16 h, as described in our pervious study reported by Ghani et al., [28]. After selfing of the allotetraploids, F_2_ allotetraploids were obtained (Additional file 8). All of the plants were grown in vermiculite mixed with 30% soil in a growth chamber under growth conditions of 22/18°C (day/night) and 16 h of illumination per day. The leaves from three plants of each type were collected 45 days after sowing in the vegetative stage for the analyses of the genetic and epigenetic alterations.

Sequence-related amplified polymorphism (SRAP) analysis was performed in the present study using twenty primer pairs (Additional file 2) according to a previously described method [57]. A modified version of the CTAB method was used to extract the genomic DNA [58]. To obtain reproducible and clear banding patterns, each amplification was repeated three times, and only bands showing consistent amplifications were scored.

### MSAP analysis

Methylation-sensitive amplification polymorphism (MSAP) analysis was performed as described by Xiong *et* al. [59]. It contains double enzymes restriction (EcoRI, HpaII/MspI), adapter ligation, pre-amplification and selective amplification. The primers used for selective amplification contained three selective nucleotides (Additional file 2). For the data analysis, values of 1 and 0 were used to represent the appearance of a fragment and the disappearance of a fragment in MSAP, respectively. Further, type (1,1) represented the appearance of fragments in both the *Msp*I and *Hpa*II lanes, type (1,0) represented the appearance of fragments only in the *Hpa*II lanes, type (0,1) represented the appearance of a fragment only in the *Msp*I lane, and type (0,0) represented the disappearance of fragments in both the *Msp*I and *Hpa*II lanes. To obtain reproducible and clear banding patterns, each amplification was repeated three times, and only bands showing consistent amplifications were scored.

### High-throughput sequencing of small RNAs

To determine small RNA populations in *B. rapa, B. nigra* and their progenitors (both the allodiploid and the allotetraploid), small RNA libraries were generated from the leaves of the four genotypes, i.e., *B. rapa* (AA), *B. nigra* (BB), the allodiploid (AB), and the allotetraploid (AABB). The total RNA was isolated using the Trizol reagent (Invitrogen, Carlsbad, CA, USA) according to the manufacturer’s instructions and was sent to Beijing Genomics Institute (BGI) for sequencing. After treatment of the raw data, the clean sequences were subjected to further analyses as previously described [60]. The clean reads were analysed by length distribution and common sequences. The sequences were then matched to the genome of all of the plants to identify the repeat-associated sRNAs and to assess the expression of sRNAs. We identified known miRNAs using miRBase. To reveal the differential expression of miRNAs, the abundance of miRNAs in all of the libraries was normalised. The normalisation values were compared between the two libraries and were calculated in the form of fold-changes (fold-change = log2 (treatment/control). Moreover, the *p-value* was obtained using a previously described formula [61]. For the prediction of targets, the gene function, including the biological process, cellular component localisation, and molecular function of the genes were analysed. The small RNA sequence data are deposited in GEO with accession number GSE61872 (http://www.ncbi.nlm.nih.gov/geo).

### The expression of miRNAs and targeted genes using quantitative RT-PCR analysis

Stem-loop qRT-PCR was used to quantify the miRNA shown to have a significantly different expression. The sequences of nine miRNAs were obtained from high-throughput sequencing, and primers were designed using the Primer software. Next, 2 μg of the total RNA were converted into cDNA using complementary designed primers. Moreover, poly (A)-tailed qRT-PCR was used to quantify the expression of the target genes. Two μg of the total RNA were converted into cDNA using oligo (dT) primers. The specific forward and reverse primers were designed using GenScript. A total volume of 25 μl containing 12.5 μl of SYBR, 2.0 μl of cDNA, 1.0 μl of the forward primer, 1.0 μl of the reverse primer, and 8.5 μl of sterilised distilled water was amplified using ABI STEPONE Real-Time PCR. The cycling conditions were 95°C for 30 s followed by 40 cycles of 5 s at 95°C and 30 s at 60°C. All of the reactions were performed in triplicate, and the U6 gene and 25 s rRNA served as references for the quantification of miRNAs and the target genes, respectively. The primers used in this study are shown in Additional file 2. The threshold cycle (CT) values were obtained automatically using ABI STEPONE, and the fold changes for each gene were quantified as the relative quantity (RQ) values using CT (2-ΔΔCt).

### Availability of supporting data

The sequences data sets supporting the results of this article are available in GEO with accession number GSE61872 (http://www.ncbi.nlm.nih.gov/geo).

## Additional files

Additional file 1: Table S2. DNA methylation levels in the parents and their allodiploid and allotetraploid plants, as detected by the methylation-sensitive amplified polymorphism (MSAP) assay. Table S3 A comparison of DNA methylation patterns based on the MSAP data between parents and their allodiploids and allotetraploids. Figure S3 DNA methylation in the parents (AA and BB) and their allodiploid (AB) and allotetraploid (AABB): (a) demethylation in all of the samples, (b) methylation in all of the samples, (c) demethylation in the offspring compared with the parents, and (d) methylation in the offspring compared with the parents. Additional file 2:
**An Excel spreadsheet containing the primer of SRAP, the sequence of the MSAP adapters and primers used in this study, the primer sequence of the miRNA, and the primer sequence of the targeted gene.**
Additional file 3: Table S1. Genetic alterations in the parents and their allodiploid and allotetraploid plants. Figure S2 Genetic alteration in the parents (AA and BB), the allodiploid (AB), and the allotetraploid (AABB): (a) novel fragments, (b) loss of fragments compared with the parents, and (c, d) parents appear to fragment with offspring. Additional file 4: Table S4. The summary of small RNA sequencing data. Table S5 The interaction between parents and their allodiploid and allotetraploid plants. Table S6 The distribution of the genome-mapped sequence reads in the small RNA libraries. Additional file 5:
**An Excel spreadsheet containing the repeat AA, BB, AB, and AABB.**
Additional file 6:
**An Excel spreadsheet containing the known miRNA expression levels between the allodiploid and allotetraploid plants, the known miRNA expression levels between the parents and the allotetraploid, the known miRNA expression levels between the parent and the allotetraploid the known miRNA expression levels between the parents and the allodiploid, the known miRNA expression levels between the parents and the allodiploid, the known miRNA expression levels between the parents, and the novel miRNA expression levels between the allodiploid and the allotetraploid, the novel miRNA expression levels between the parents and the allotetraploid, the novel miRNA expression levels between the parents and the allotetraploid, the novel miRNA expression levels between the parents and the allodiploid, the novel miRNA expression levels between the parents and the allodiploid, the novel miRNA expression levels between the parents, and the known miRNA sequence AABB, the known miRNA sequence AB, the known miRNA sequence AA, the known miRNA sequence BB, the novel miRNA sequence AABB, the novel miRNA sequence AB, the novel miRNA sequence AA, the novel miRNA sequence BB, and the known miRNAs and prediction target genes in AA, the known miRNAs and prediction target genes in BB, the known miRNAs and prediction target genes in AB, the known miRNAs and prediction target genes in AABB, and the novel miRNAs and prediction target genes in AA, the novel miRNAs and prediction target genes in BB, the novel miRNAs and prediction target genes in AB and the novel miRNAs and prediction target genes in AABB.**
Additional file 7:
**An Excel spreadsheet containing the related crop family miRNAs.**
Additional file 8: Figure S1. The number of days for the flowering of the parents and their allodiploid and allotetraploid plants.
