# Supplementary material for: The role of small RNAs in wide hybridisation and allopolyploidisation between Brassica rapa and Brassica nigra
Source: BMC Plant Biol. 2014 Oct 19;14:272. doi: 10.1186/s12870-014-0272-9 (PMC4209033; doi:10.1186/s12870-014-0272-9)
Supplement: Additional file 1: Table S2. — DNA methylation levels in the parents and their allodiploid and allotetraploid plants, as detected by the methylation-sensitive amplified polymorphism (MSAP) assay. Table S3 A comparison of DNA methylation patterns based on the MSAP data between parents and their allodiploids and allotetraploids. Figure S3 DNA methylation in the parents (AA and BB) and their allodiploid (AB) and allotetraploid (AABB): (a) demethylation in all of the samples, (b) methylation in all of the samples, (c) demethylation in the offspring compared with the parents, and (d) methylation in the offspring compared with the parents. [file 12870_2014_272_MOESM1_ESM.doc]

**Table S2** DNA methylation levels in the parentsand their allodiploid and allotetraploid, as detected by the methylation-sensitive amplified polymorphism (MSAP) assay.

| Sample | DNA methylation status | | | | Total bands | Total methylated bands | Total methylated % |
| --- | --- | --- | --- | --- | --- | --- | --- |
|  | 11 | 10 | 01 | 00 |  |  |  |
| AA | 425 | 254 | 304 | 471 | 1449 | 1029 | 71.01^b^ |
|  | 29.33 % | 17.52% | 20.97 % | 32.51 % |  |  |  |
| BB | 384 | 329 | 305 | 431 | 1449 | 1065 | 73.50^a^ |
|  | 26.50 % | 22.71 % | 21.05 % | 29.74 % |  |  |  |
| AB | 451 | 231 | 355 | 412 | 1449 | 998 | 68.88^c^ |
|  | 31.12 % | 15.94 % | 24.50 % | 28.43 % |  |  |  |
| AABB | 568 | 207 | 270 | 404 | 1449 | 881 | 60.80^d^ |
|  | 39.20 % | 14.29 % | 18.63 % | 27.87 % |  |  |  |

**Table S3** Comparison of DNA methylation patterns based on the MSAP data between parents and their allodiploid and allotetraploid.

| Patterns | AA | BB | Hybrids (AB or AABB) | No. and ratio of  Patterns (AA,BB,AB) | | Methylation in hybrids | No. and ratio of  Patterns (AA,BB,AABB) | |
| --- | --- | --- | --- | --- | --- | --- | --- | --- |
|  | HM | HM | HM | No. | Ratio |  | No. | Ratio |
| a | 11 | 11 | 11 | 177 | 12.22 | ○ | 175 | 12.08 |
| b | 10 | 10 | 10 | 78 | 11.94 | ● | 78 | 11.94 |
|  | 01 | 01 | 01 | 95 |  | ● | 95 |  |
|  |  |  |  | 173 |  | ● | 173 |  |
| c | 11 | 00 | 11 | 90 | 18.91 | ○ | 104 | 28.64 |
|  | 11 | 10 | 11 | 19 |  | ○ | 23 |  |
|  | 11 | 01 | 11 | 14 |  | ○ | 15 |  |
|  | 10 | 00 | 11 | 1 |  | ○ | 3 |  |
|  | 10 | 11 | 11 | 11 |  | ○ | 6 |  |
|  | 10 | 10 | 11 | 7 |  | ○ | 20 |  |
|  | 10 | 01 | 11 | 4 |  | ○ | 3 |  |
|  | 01 | 01 | 11 | 4 |  | ○ | 8 |  |
|  | 01 | 11 | 11 | 21 |  | ○ | 21 |  |
|  | 01 | 00 | 11 | 12 |  | ○ | 16 |  |
|  | 01 | 10 | 11 | 2 |  | ○ | 2 |  |
|  | 00 | 11 | 11 | 64 |  | ○ | 49 |  |
|  | 00 | 00 | 11 | 2 |  | ○ | 8 |  |
|  | 00 | 10 | 11 | 1 |  | ○ | 32 |  |
|  | 00 | 01 | 11 | 14 |  | ○ | 22 |  |
|  | 00 | 11 | 11 | 8 |  | ○ | 61 |  |
|  |  |  |  | 274 |  | ○ | 415 |  |
| d | 11 | 11 | 10 | 3 | 56.94 | ● | 3 | 48.10 |
|  | 11 | 11 | 01 | 5 |  | ● | 6 |  |
|  | 11 | 11 | 00 | 8 |  | ● | 1 |  |
|  | 11 | 10 | 10 | 19 |  | ● | 17 |  |
|  | 11 | 10 | 01 | 4 |  | ● | 1 |  |
|  | 11 | 10 | 00 | 5 |  | ● | 6 |  |
|  | 11 | 01 | 10 | 2 |  | ● | 1 |  |
|  | 11 | 01 | 01 | 10 |  | ● | 9 |  |
|  | 11 | 01 | 00 | 2 |  | ● | 2 |  |
|  | 11 | 00 | 10 | 26 |  | ● | 25 |  |
|  | 11 | 00 | 01 | 22 |  | ● | 13 |  |
|  | 11 | 00 | 00 | 34 |  | ● | 30 |  |
|  | 10 | 10 | 01 | 2 |  | ● | 5 |  |
|  | 10 | 10 | 00 | 39 |  | ● | 43 |  |
|  | 10 | 11 | 10 | 3 |  | ● | 2 |  |
|  | 10 | 11 | 01 | 6 |  | ● | 11 |  |
|  | 10 | 11 | 00 | 3 |  | ● | 4 |  |
|  | 10 | 01 | 01 | 3 |  | ● | 3 |  |
|  | 10 | 00 | 10 | 21 |  | ● | 15 |  |
|  | 10 | 00 | 00 | 71 |  | ● | 74 |  |
|  | 01 | 11 | 01 | 4 |  | ● | 3 |  |
|  | 01 | 11 | 00 | 4 |  | ● | 3 |  |
|  | 01 | 10 | 10 | 1 |  | ● | 1 |  |
|  | 01 | 10 | 01 | 3 |  | ● | 3 |  |
|  | 01 | 10 | 00 | 7 |  | ● | 7 |  |
|  | 01 | 01 | 00 | 4 |  | ● | 6 |  |
|  | 01 | 01 | 01 | 7 |  | ● | 0 |  |
|  | 01 | 00 | 10 | 4 |  | ● | 9 |  |
|  | 01 | 00 | 01 | 35 |  | ● | 39 |  |
|  | 01 | 00 | 00 | 86 |  | ● | 73 |  |
|  | 00 | 11 | 10 | 8 |  | ● | 5 |  |
|  | 00 | 11 | 01 | 26 |  | ● | 26 |  |
|  | 00 | 11 | 00 | 33 |  | ● | 45 |  |
|  | 00 | 10 | 10 | 49 |  | ● | 34 |  |
|  | 00 | 10 | 01 | 1 |  | ● | 3 |  |
|  | 00 | 10 | 00 | 92 |  | ● | 72 |  |
|  | 00 | 01 | 10 | 1 |  | ● | 2 |  |
|  | 00 | 01 | 01 | 121 |  | ● | 38 |  |
|  | 00 | 01 | 00 | 24 |  | ● | 38 |  |
|  | 00 | 00 | 10 | 16 |  | ● | 15 |  |
|  | 00 | 00 | 01 | 11 |  | ● | 15 |  |
|  |  |  |  | 825 |  |  | 697 |  |
| Total |  |  |  | 1449 |  |  | 1449 |  |

The open circle (○) indicates demethylation in the hybrid compared to one or both of the parents. The filled circle (●) indicates methylation or hypermethylation in the hybrid compared to one or both of the parents.


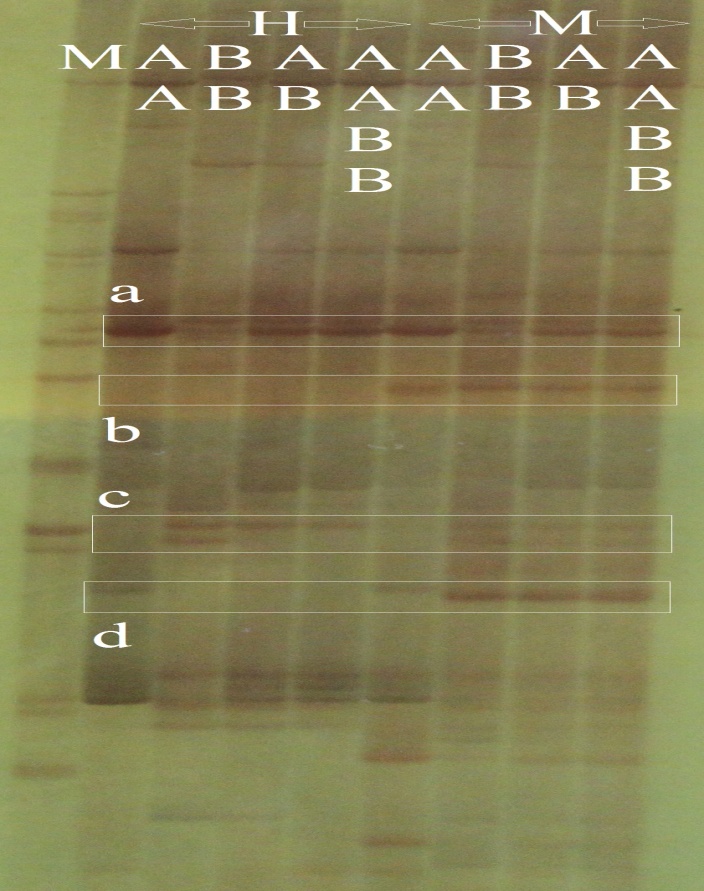


**Figure S3** DNA methylation in the parents (AA and BB) and their allodiploid (AB) and allotetraploid (AABB): (a) demethylation in all of the samples, (b) methylation in all of the samples, (c) demethylation in the offspring compared with the parents, and (d) methylation in the offspring compared with the parents.
