# Supplementary material for: The role of small RNAs in wide hybridisation and allopolyploidisation between Brassica rapa and Brassica nigra
Source: BMC Plant Biol. 2014 Oct 19;14:272. doi: 10.1186/s12870-014-0272-9 (PMC4209033; doi:10.1186/s12870-014-0272-9)
Supplement: Additional file 3: Table S1. — Genetic alterations in the parents and their allodiploid and allotetraploid plants. Figure S2 Genetic alteration in the parents (AA and BB), the allodiploid (AB), and the allotetraploid (AABB): (a) novel fragments, (b) loss of fragments compared with the parents, and (c, d) parents appear to fragment with offspring. [file 12870_2014_272_MOESM3_ESM.doc]

**Table S1** Genetic alterations in parents and their allodiploid and allotetraploid

| Sample | Total fragments | Appear fragments with parents | | Disappear fragments with parents | | Novel fragments | Total genetic alteration | Genetic alteration |
| --- | --- | --- | --- | --- | --- | --- | --- | --- |
|  |  | H | NY | H | NY |  |  | % |
| AA | 350 | − | − | − | − | − | − | − |
| BB | 401 | − | − | − | − | − | − | − |
| AB | 455 | 22 | 36 | 12 | 16 | 19 | 47 | 10.32^b^ |
| AABB | 495 | 44 | 72 | 27 | 38 | 41 | 106 | 21.41^a^ |

0000000000000000000000000000000000000000000000000000000000000000000000000000000000000000000000000000000000000000000000000000000


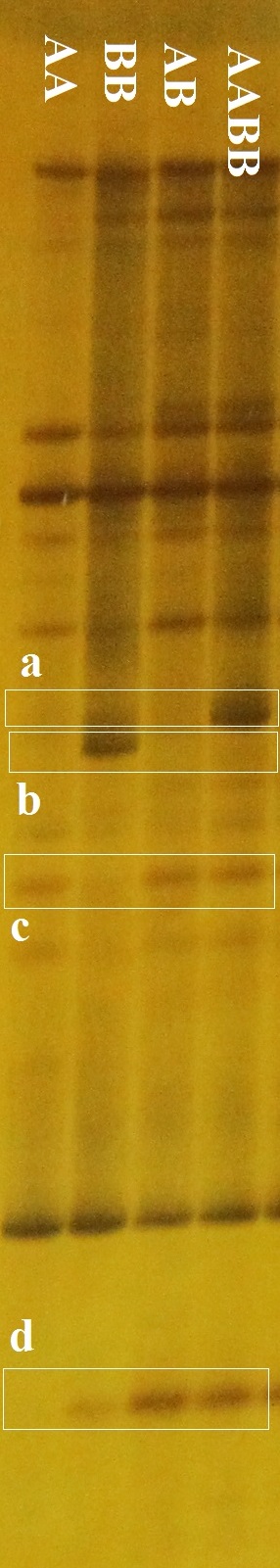


**Figure S2** Genetic alteration in the parents (AA and BB), allodiploid (AB), and allotetraploid (AABB): (a) novel fragments, (b) loss of fragments compared with the parents, and (c, d) parent appear fragment with offspring.
