# Supplementary material for: The role of small RNAs in wide hybridisation and allopolyploidisation between Brassica rapa and Brassica nigra
Source: BMC Plant Biol. 2014 Oct 19;14:272. doi: 10.1186/s12870-014-0272-9 (PMC4209033; doi:10.1186/s12870-014-0272-9)
Supplement: Additional file 4: Table S4. — The summary of small RNA sequencing data. Table S5 The interaction between parents and their allodiploid and allotetraploid plants. Table S6 The distribution of the genome-mapped sequence reads in the small RNA libraries. [file 12870_2014_272_MOESM4_ESM.doc]

**Table S4 Summary of small RNA sequencing data**

| Type | AA |  | BB |  | AB |  | AABB |
| --- | --- | --- | --- | --- | --- | --- | --- |
|  | count |  | count |  | count |  | count |
| total reads | 10654462 |  | 10284599 |  | 10916518 |  | 9954925 |
| high quality | 10617193 (100%) |  | 10248915 (100%) |  | 10877646 (100%) |  | 9921069 (100%) |
| 3'adapter null | 1693 (0.02%) |  | 1714 (0.02%) |  | 2647 (0.02%) |  | 1541 (0.02%) |
| insert null | 2852 (0.03%) |  | 1877 (0.02%) |  | 3136 (0.03%) |  | 1632 (0.02%) |
| 5'adapter contaminants | 34591 (0.33%) |  | 75528 (0.74%) |  | 159731 (1.47%) |  | 36899 (0.37%) |
| Smaller than 18nt | 56390 (0.53%) |  | 65119 (0.64%) |  | 195649 (1.80%) |  | 76960 (0.78%) |
| polyA | 666 (0.01%) |  | 798 (0.01%) |  | 762 (0.01%) |  | 1021 (0.01%) |
| clean reads | 10521976 (99.10%) |  | 10102904 (98.58%) |  | 10515721 (96.67%) |  | 9803016 (98.81%) |

**Table S5 Interaction between parents and their allodiploid and allotetraploid**

| Class | Unique sRNAS | Percent (%) | Total sRNAS | Percent (%) |
| --- | --- | --- | --- | --- |
| Total sRNAs of AA & BB | 4050500 | 100.00% | 20624880 | 100.00% |
| AA & BB | 160382 | 3.96% | 12517811 | 60.69% |
| AA specific | 1874552 | 46.28% | 4011714 | 19.45% |
| BB specific | 2015566 | 49.76% | 4095355 | 19.86% |
| Total sRNAs of AA & AB | 3753616 | 100.00% | 21037697 | 100.00% |
| AA & AB | 575751 | 15.34% | 17106237 | 81.31% |
| AA specific | 1459183 | 38.87% | 1696152 | 8.06% |
| AB specific | 1718682 | 45.79% | 2235308 | 10.63% |
| Total sRNAs of AA & AABB | 3958365 | 100.00% | 20324992 | 100.00% |
| AA & AABB | 417947 | 10.56% | 15197850 | 74.77% |
| AA specific | 1616987 | 40.85% | 2185445 | 10.75% |
| AABB specific | 1923431 | 48.59% | 2941697 | 14.47% |
| Total sRNAs of BB & AB | 4311021 | 100.00% | 20618625 | 100.00% |
| BB & AB | 159360 | 3.70% | 12102607 | 58.70% |
| BB specific | 2016588 | 46.78% | 4080098 | 19.79% |
| AB specific | 2135073 | 49.53% | 4435920 | 21.51% |
| Total sRNAs of AABB & BB | 4165902 | 100.00% | 19905920 | 100.00% |
| AABB & BB | 351424 | 8.44% | 13536377 | 68.00% |
| AABB specific | 1989954 | 47.77% | 3598711 | 18.08% |
| BB specific | 1824524 | 43.80% | 2770832 | 13.92% |
| Total sRNAs of AABB & AB | 4218624 | 100.00% | 20318737 | 100.00% |
| AABB & AB | 417187 | 9.89% | 14616941 | 71.94% |
| AABB specific | 1924191 | 45.61% | 2947673 | 14.51% |
| AB specific | 1877246 | 44.50% | 2754123 | 13.55% |

**Table S6 Distribution of the genome-mapped sequence reads in small RNA libraries**

| Category | AA | | BB | | AB | | AABB | |
| --- | --- | --- | --- | --- | --- | --- | --- | --- |
|  | Unique sRNAs | Total sRNAs | Unique sRNAs | Total sRNAs | Unique sRNAs | Total sRNAs | Unique sRNAs | Total sRNAs |
| Total | 2034934  (100%) | 10521976  (100%) | 2175948  (100%) | 10102904  (100%) | 2294433  (100%) | 10515721  (100%) | 2341378  (100%) | 9803016  (100%) |
| Exon antisense | 3138  (0.15%) | 6180  (0.06%) | 1780  (0.08%) | 4644  (0.05%) | 2832  (0.12%) | 5086  (0.05%) | 2422  (0.10%) | 3816  (0.04%) |
| Exon sense | 16252  (0.80%) | 32593  (0.31%) | 15032  (0.69%) | 34540  (0.34%) | 15863  (0.69%) | 32059  (0.30%) | 10630  (0.45%) | 19238  (0.20%) |
| Intron antisense | 200  (0.01%) | 269  (0%) | 140  (0.01%) | 240  (0%) | 165  (0.01%) | 214  (0%) | 234  (0.01%) | 397  (0%) |
| Intron sense | 698  (0.03%) | 889  (0.01%) | 417  (0.02%) | 512  (0.01%) | 612  (0.03%) | 754  (0.01%) | 490  (0.02%) | 591  (0.01%) |
| miRNA | 22954  (1.13%) | 2526482  (24.01%) | 22716  (1.04%) | 2176470  (21.54%) | 24143  (1.05%) | 2183129  (20.76%) | 23061  (0.98%) | 2524964  (25.76%) |
| rRNA | 180332  (8.86%) | 2677435  (25.45%) | 184445  (8.48%) | 2717179  (26.90%) | 170676  (7.44%) | 2570703  (24.45%) | 126458  (5.40%) | 1514595  (15.45%) |
| repeat | 1270  (0.06%) | 42307  (0.40%) | 1532  (0.07%) | 17258  (0.17%) | 1628  (0.07%) | 63114  (0.60%) | 2084  (0.09%) | 155977  (1.59%) |
| snRNA | 2328  (0.11%) | 7378  (0.07%) | 1825  (0.08%) | 5046  (0.05%) | 2121  (0.09%) | 5468  (0.05%) | 2173  (0.09%) | 4812  (0.05%) |
| snoRNA | 1167  (0.06%) | 2599  (0.02%) | 897  (0.04%) | 1705  (0.02%) | 920  (0.04%) | 1631  (0.02%) | 893  (0.04%) | 1667  (0.02%) |
| tRNA | 16627  (0.82%) | 510445  (4.85%) | 16433  (0.76%) | 676355  (6.69%) | 15368  (0.67%) | 515949  (4.91%) | 11700  0(.50%) | 327739  (3.34%) |
| unann | 1789968  (87.96%) | 4715399  (44.81%) | 1930731  (88.73%) | 4468955  (44.23%) | 2060105  (89.79%) | 5137614  (48.86%) | 2161233  (92.31%) | 5249220  (53.55%) |
