# Supplementary material for: The role of small RNAs in wide hybridisation and allopolyploidisation between Brassica rapa and Brassica nigra
Source: BMC Plant Biol. 2014 Oct 19;14:272. doi: 10.1186/s12870-014-0272-9 (PMC4209033; doi:10.1186/s12870-014-0272-9)
Supplement: Additional file 8: Figure S1. — The number of days for the flowering of the parents and their allodiploid and allotetraploid plants. [file 12870_2014_272_MOESM8_ESM.doc]

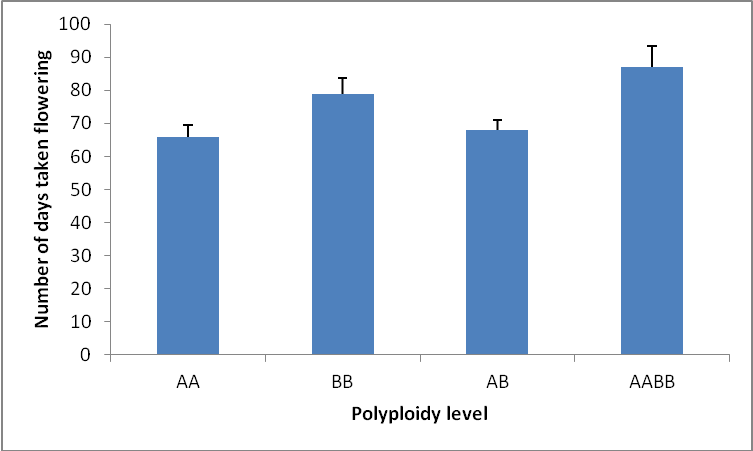


**Figure S1** Number of days for the flowering of the parents and their allodiploid and allotetraploid plants.
